# The Impact of Hormonal Imbalances Associated with Obesity on the Incidence of Endometrial Cancer in Postmenopausal Women

**DOI:** 10.7150/jca.47580

**Published:** 2020-07-11

**Authors:** Sarah Ding, Chikezie O. Madu, Yi Lu

**Affiliations:** 1 Departments of Clinical and Diagnostic Sciences, University of Alabama at Birmingham, Birmingham, AL 35294, USA.; 2 Departments of Biological Sciences, University of Memphis, Memphis, TN 38152, USA.; 3 Department of Pathology and Laboratory Medicine, University of Tennessee Health Science Center, Memphis, TN 38163, USA.

**Keywords:** endometrial cancer, adipokines, estrogen, insulin and insulin-like factors, leptin, visfatin, adiponectin

## Abstract

Obesity has long been associated with endometrial cancer amongst postmenopausal women; in fact, obese women are more than twice as likely to develop endometrial cancer as women of normal weight. The risk of developing this type of cancer increases with weight gains in adulthood, especially among women who did not use hormonal therapy for menopause. Thus, with an association between menopause, obesity, and endometrial cancer established, it prompts the following question: what specific factors could cause higher risk levels for endometrial cancer in this cohort of women? In this paper, the factor of hormonal changes and imbalances associated with both obesity and menopause will be examined. The hormones that will be discussed are insulin and insulin-like factors, estrogen, and adipokines (specifically adiponectin, visfatin, and leptin).

## Introduction

Endometrial cancer (EC), also referred to as uterine cancer, is characterized by tumors and lesions found in the lining of the uterus in many older women. Symptoms may include vaginal bleeding and pelvic pain, though some women report no symptoms. It is divided into type I and type II EC, each with distinct physical and molecular differences. Type I is most common in post-menopausal women and is often associated with obesity 1. They typically make up about 85% of EC cases and are glandular in structure; they also express higher levels of estrogen receptors (in particular estrogen receptor alpha) and are postulated to be primarily hormonally derived. Type I cases are most often associated with obesity 2. Meanwhile, Type II EC is much rarer (making up the remaining 15%) and is believed to be rooted deeper in DNA error. Cases can be found in both pre-menopausal and post-menopausal; many cases involve copy number variation and p53 mutations. Pathophysiology of the development of Type II EC involves changes to the function of aforementioned genes such as p53 (inhibition to the apoptosis of genetically dysfunctional cells), as well as modifications of oncogenes such as P13K, Akt, etc 3. Among women, EC ranks as the fourth most common type of cancer with a declining five-year survival rate and a rising rate of occurrence; thus, being able to effectively prevent, diagnose, and treat this cancer has become more imperative as time passes.

One factor found to be linked to a higher incidence of EC, particularly Type I EC, is obesity, defined as having a body mass index of thirty or higher 4. The development of obesity follows a sequence wherein adipose tissue develops, followed by the growth of older adipocytes and the proliferation and differentiation of newer adipocytes 5. This increase in adipose tissue has several implications for the hormonal balance of the body, as adipose tissue is itself a complex and highly active endocrine organ that participates extensively in many aspects of bodily function, such as nutrient homeostasis and energy storage 6. One of obesity's main links to the problem of cancer is that it usually involves an increase in visceral fat, which leads to significantly increased insulin hormonal activity. This onset of visceral fat can be augmented by the onset of menopause wherein the lowered levels of circulating estrogen may lead to a loss of subcutaneous fat and an increase in visceral fat, for estrogen has been linked to the accumulation of subcutaneous fat 7. This change in fat distribution implicates a major role for estrogen in the development of post-menopausal obesity, making it a candidate for examination in the discussion of the development of EC.

Adipose tissue is also a major source of small peptide growth hormone factors known as adipokines, primarily secreted from white adipose tissue. These hormones have a range of effects, from anti-inflammatory effects such as adiponectin to promoting angiogenesis and cell proliferation, as is the case with leptin 8. Adipose tissue is also known to be a source of estrogen through a process of peripheral site synthesis wherein androgens produced by the adrenal cortex and a postmenopausal ovary are converted into estrogen through the assistance of an enzyme called aromatase, primarily found in adipose tissue in areas such as the breast. In fact, the production of estrogen through peripheral site synthesis is so prolific that a popular theory in the research of breast cancer postulates that this mechanism of production allows concentrations of estrogens within the breast to increase up to tenfold higher than the circulating level, which in turn plays a role in the development of breast cancer 9. As such, an increase in the quantity and distribution of adipose tissue in women following menopause leads to a drastic shift in the production of these hormones. Such an increase is subject to scrutiny, as it affects their circulating concentrations and potentially exerts effects on the development of EC.

In this paper, the correlation between obesity and menopause and the occurrence of EC will be examined through an investigation of the hormones associated with each condition. In the past decades, it has been shown that there exists a strong correlation between these two factors and EC. The components associated with the endocrine system of each of these conditions will be traced and their influence upon the incidence of EC discussed. In the text, there will be a breakdown of the primary hormones that are observed to be prominent factors in the onset of EC in postmenopausal women experiencing obesity: first, insulin and insulin-like factors; then estrogen; and finally, adipokines, further divided into adiponectin, leptin, and visfatin.

## Insulin and Insulin-Like Factors

It has been established that obesity often raises the blood levels of insulin and insulin-like growth factors, for as BMI increases there is also a direct increase in circulating insulin levels 10. While insulin levels do not reveal a strong correlation with menopause or post-menopausal hormone therapy (thereby establishing no strong direct link with estrogen levels), the trend in increasing abdominal and visceral adipose tissue accumulation associated with menopause points to an indirect increase in insulin levels through this accumulation of adipose tissue. As such, the increase in insulin associated with both of these conditions has been cited as a factor in the development of cancer.

### Insulin

Obesity occurs through enlarged adipocyte cells as well as through an increase in the number of these adipose cells, both processes that are regulated by insulin levels. Insulin is a mitogen with antiapoptotic activity, something of particular concern since many EC cell lines express high-affinity insulin receptors 10. Malfunctioning adipocytes are closely linked to insulin-resistance associated with obesity as well through the release of pro-inflammatory adipokines that may act by disrupting crucial signaling pathways 11. This incidence of malfunctioning adipocytes is enhanced by the onset of obesity due to the sheer higher volume of adipocytes. Insulin resistance, in turn, produces higher levels of blood insulin as the body continues to secrete insulin in an attempt to maintain homeostasis in a malfunctioning negative feedback loop. Research shows that the expression of insulin-receptor subunits, especially the ligand-binding domain, increases the growth of EC cells. Cells taken from endometrial carcinomas also demonstrate much higher levels of these two insulin-receptor subunits than do other forms of carcinoma 11. Insulin has also been shown to promote the development of tumors through activating ras-raf-MAPK pathways and other pathways that promote cell survival 12.

### Insulin-like Growth Factors (IGF-1)

Insulin-like growth factors (IGF), though mainly secreted by the liver, can be secreted locally at varying levels throughout the body; they act as cell cycle progression factors, allowing the cell to traverse G0/G1 phases, by activating phosphatidylinositol-3-kinase (PI-3K)/Akt signaling cascade and mitogen-activated protein kinase (MAPK) 5. Impairment of this signaling pathway may lead to activation of other factors that extend cell lifespan, while over-activation of the pathway may induce an accelerated aging process. IGFs are also pivotal in coordinating protein, carb, and fat metabolism. Its synthesis is stimulated by nutrition and growth hormone (GH) in the liver; through reducing the metabolic effects of GH, IGF can increase insulin sensitivity, contrary to GH's role of inhibiting insulin signaling in muscle and at cells. It is also worth noting that a deficiency in GH led to a relatively larger visceral adiposity which may serve to amplify the effects of excess adipose tissue, study shows 13. In obesity, an increase in IGF-1 has been recorded to lead to an increase in GH binding protein levels, thereby resulting in a lack of suppression of IGF-1. Insulin-like growth factors are also shown to play an active role in the development of EC, partially through the activity of insulin levels and resistance brought on by obesity and adipose fat accumulation associated with menopause.

IGF-1 binds to insulin-like growth factor cell receptors (IGF1R), which are one of the two classes of tyrosine kinase receptors primarily involved in anti-apoptotic and transforming activities in the area. This receptor has been shown to be crucial in preadipocyte differentiation and proliferation and overall survival 13. Its mechanism primarily involves binding to IGF to initiate the ones described previously. Since high IGF1R expression is consistent across all gynecological cancers, it has been attributed to as much as 91.3% of all ECs 12. In addition, it has been found that overexpression of the IFG1R gene is associated with the initiation of oncogenic transformation in many of these cancers; however, due to the nature of other hormones, the level of expression of IGF1R and IG differs between stages and types of cancer, which must be kept in consideration as research continues concerning the IGF complex. Despite this, IGF1R has been shown to be essential for survival, as shown by a lethal phenotype in mice where the IGF1R gene was disrupted 14.

IGF-1 levels are reduced by the presence of IGFBP-1 (IGF binding protein-1; of the six classes of IGFBP, this one is the most widely studied and thus will be the main focus of this section) since this binding protein directly competes with IGF-1 for binding sites; thus, the levels of bioavailable IGF-1 in the body are regulated by the balance of IGF-1 and IGFBP-1 expression. However, in the case of EC, estrogen (a major hormone in the development of EC) increases IGF-1 expression while its counterpart, progesterone, stimulates the production of IGFBP-1, which in turn keeps IGF-1 levels in check. Insulin is also capable of indirectly increasing IGF-1 through decreasing expression of IGFBP-1 in hepatocytes 11. IGFBP levels are known to correlate inversely with measures of adiposity and insulin levels 13.

**Figure 1:**
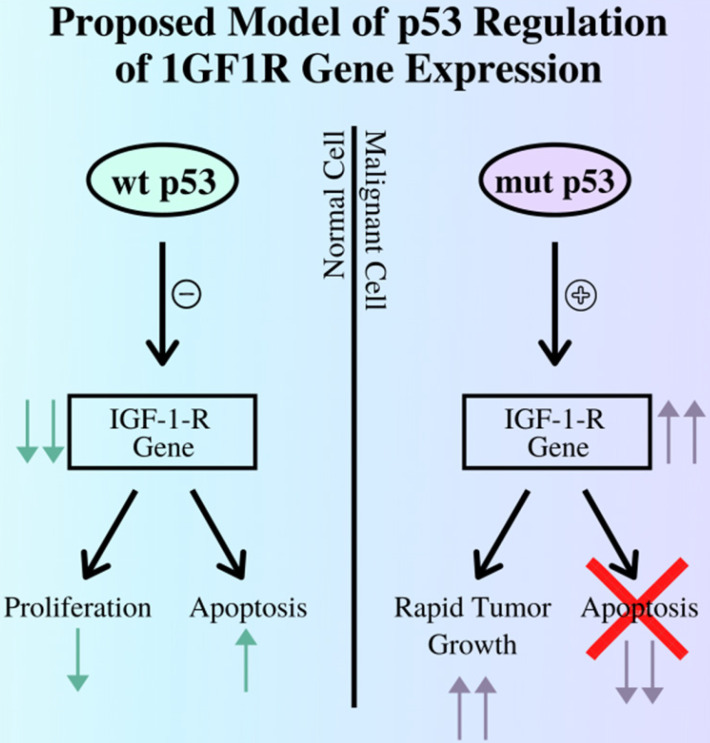
The relationship between the p53 gene and expression of the IGF-I-R gene by contrasting the outcomes of pathways regulated by functioning, wild type p53 gene and mutant, non-functional p53 gene. As demonstrated above a mutant p53 cannot suppress the expression of the IGF1R gene, leading to tumor growth while diverging from the apoptosis pathway 12.

The p53 gene also plays a role in the development of cancers, as its role in a healthy cell is to initiate apoptosis pathways in the case of irreparable DNA damage to prevent these errors from proliferating in the body. In relation to the IGF complex, the p53 gene regulates IGF-1R expression mainly by repressing the IGF-IR promoter 15. Thus, in cases where the p53 gene is damaged and can no longer perform its role, cancerous cells are allowed to survive and grow with no restraints, aided in part by uncontrolled IGF-1R expression, as illustrated in Figure 1. Other studies demonstrate a negative correlation between p53 expression and survival rates from EC 12. Thus, IGF1P activity increases due to the increase in cell-surface receptors, leading to further promotion of cell growth and oncogene transformation.

Both insulin and insulin-like growth factors play crucial roles in the development of EC through interactions with various other genes and pathways, such as p53. These correlations are amplified by higher levels of abdominal fat seen in obesity and post-menopause.

## Estrogen

**Figure 2:**
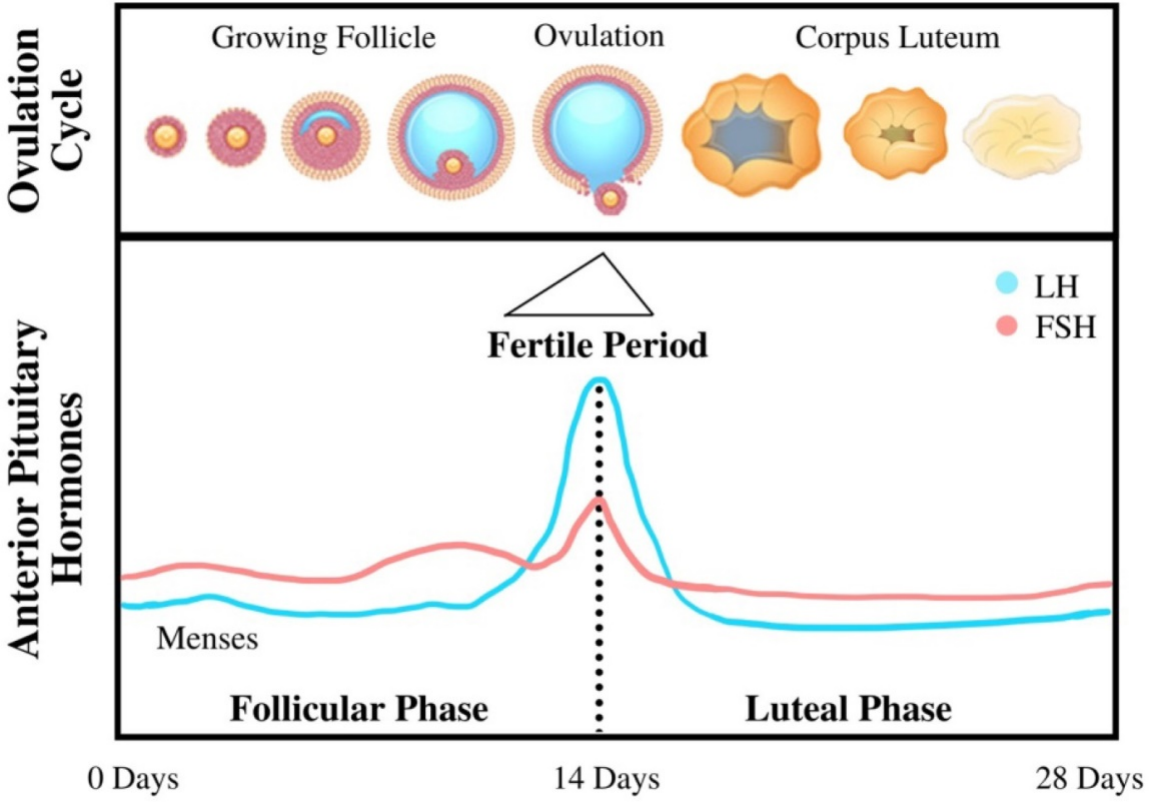
Diagram of the menstrual cycle illustrating the steps and processes of the cycle, as well as various hormone levels associated with each stage 20.

The beginning of menopause is characterized by loss of function in female reproductive organs in regulating bodily levels of endogenous hormones, such as estrogen, progesterone, and testosterone. This loss of control is caused by decreasing responsiveness to Follicle-Stimulating Hormone and Luteinizing Hormone (as shown in Figure 2), two hormones that are essential in reproduction 16. In healthy premenopausal women, estrogen is mainly produced by the ovaries under the influence of these gonadotropins; however, a fraction of the total estrogen in the body is also produced through adipose tissue through a process called aromatization whereby androgens secreted by the pituitary gland and postmenopausal ovaries are converted to estrogen through a complex of enzymes called aromatase 17. Adipokines (primarily TNFα and IL-6) secreted by adipose tissue may also induce signaling pathways that further increase the production of aromatase, thereby amplifying the secretion of estrogen 18. Menopause is usually characterized by lower levels of estrogen (estrogen depletion) as a result of lower levels of gonadotropins from the pituitary gland; however, in cases of obesity combined with menopause, women are often characterized by the opposite condition—an excess of free estrogen in the body. This excess estrogen has been clearly linked to the onset of EC across multiple studies and reviews. The excess of peripherally produced estrogen is amplified with an increase in visceral adipose tissue frequently associated with both menopause and obesity: as fat mass increases with increasing body weight, aromatase expression—and thus free estrogen levels—also increases 17. Estrogen's connection to the occurrence of EC has been confirmed by a number of case studies, such as those that indicate EC patients have increased peripheral estrogen conversion—up to four times greater than that of the young adult or the patient without cancer 19, even when adjusted to account for other factors such as age and genetic background.

The mechanism linking high levels of unopposed estrogen and the occurrence of EC is still being investigated as a topic of heavy research. The prevailing theory is that EC stems from exaggerated cell proliferation that would normally occur during the menstrual cycle, thereby increasing the likelihood of genetic errors and resulting in malignant transformation. In this process, during the “proliferation” phase extensive cell growth occurs in the lining of the uterus (the epithelium) in preparation for the reception of the eggs 2. This stage is marked by high levels of estrogen and lower levels of progesterone and other androgens, which acts by inhibiting estrogen's growth signal to endometrial cells. In addition, the process also exhibits higher production of free IGF-1 factors (as a result of higher levels of estrogen) and lower levels of IGF receptors (as a result of low levels of progesterone) which also plays a significant role in enabling this cell proliferation by transforming E2 estrogen into its less potent E1 form 20. It also facilitates the conversion of free estrogen to estrogen sulfates, which take estrogen out of the plasma and excretes it from the body. This “unopposed estrogen” theory is further supported by the observation that a sharp increase in progesterone levels in the luteal phase correlates to a significant decrease in endometrial cell proliferation 2. Low progesterone levels can be exacerbated by a condition commonly associated with obesity in postmenopausal women known as anovulation, wherein a lack of ovulation and corpus luteum production keeps progesterone levels low, thus unable to suppress endometrial growth under the influence of estrogen.

The unopposed estrogen theory is further supported by the increase in cases of EC following the introduction of hormone replacement therapy utilizing unopposed estrogen in the 1960s and 70s 21. However, it must be noted that although progestins counteract estrogen in hormone replacement therapy, the manner in which they are administered also plays a major role in the overall prognosis following treatment 22.

## Adipokines

**Figure 3:**
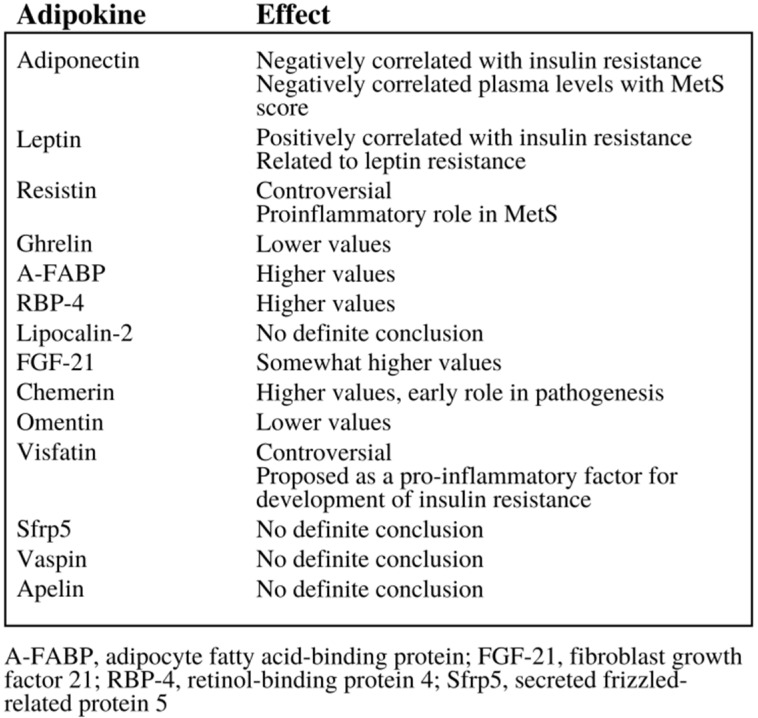
The variety of effects that adipokines can have, especially in the area of insulin resistance 6.

Adipokines are small peptide hormonal growth factors secreted by adipose tissue that play a significant growth in controlling the growth of cells, as they can both encourage and inhibit their growth, depending on the specific adipokine as demonstrated in Figure 3 23. These adipokines are often associated with metabolic disorders, hence the emphasis on adipokines' effect on insulin resistance demonstrated in Figure 3. Adipose tissue depot output differs widely based on factors such as the types of cells making up the tissue, diet, and location of the adipose tissue. Thus, the addition of excess adipose tissue associated with obesity leads to abnormalities in adipokine secretion, which in turn leads to the development of metabolic and inflammatory syndromes and even cancer 25; in fact, studies show that the increased production and secretion of a host of adipokines may influence tumor progression, especially in obese patients 8. In addition, adipokines do not only affect organs that their adipocytes are located in; they can also affect other metabolically-relevant organs, such as the liver and muscles 24. Thus, adipose tissue can even be considered the largest endocrine gland in the body, able to modulate insulin sensitivity and other factors that can potentially lead to diabetes, other metabolic syndromes, and even cancer 24.

Adipokines are reported to have a wide range of effects depending on the class of adipokines in question. Some, like leptin, are reported to activate pathways involved with EC such as mTOR. Others such as adiponectin activate AMP-activated protein kinase pathways that ultimately inhibit pathways like mTOR. Still others such as IL-6 activate pathways that promote cancer cell survival, proliferation, and metastasis. Some even act to undermine the genomic stability of key signaling proteins to indirectly influence cell proliferation 3. Research has found that an increase in the frequency of pro-inflammatory adipokines and a decrease in the frequency of anti-inflammatory adipokine is what results in the development of cardiovascular and inflammatory diseases associated with obesity.

Complications associated with obesity, such as inflammation and insulin resistance, are a result of immune cell infiltration of adipose tissue. Specifically, the accumulation of macrophages and fibroblasts increases as the weight of an individual increases 25. This accumulation leads to greater inflammation and insulin resistance, as well as metabolic dysfunction due to the restrictions of the matrix produced by fibroblasts. The activation of macrophages has been linked to the presence of specific T-cells inhabiting the tissue. These T-cells stimulate a pro-inflammatory phenotype of the macrophage, thus resulting in inflammation.

With respect to EC, individual adipokines have varying effects on its development; their levels also differ with the onset of obesity. In the next few sections, the effects of specific adipokines will be examined.

### Leptin

Leptin is a 16-kDa polypeptide hormone produced by adipocytes within fat tissue, typically white adipose tissue. It acts primarily as the ligand to its receptor, Ob-R, which is thought to promote tumor manifestation through activation of PI3K, MAPK, and STAT3 pathways via phosphorylation of downstream transcription factor of STAT and autophosphorylation of JAK1 and JAK2. In monitoring energy stores, leptin typically acts by binding to receptors (LEPR-B) in the hypothalamus of the cerebral ventricle in the brain 26. LEPR-B acts by stimulating the JAK-STAT signal transduction pathway. This pathway leads to the development of two classes of neuropeptides within the hypothalamus: orexigenic (appetite-inducing) and anorexic (appetite-suppressing). Leptin signaling is inhibited by a family of proteins known as SOCS proteins, which are induced by cytokines and exert a regulatory influence over leptin via a negative feedback loop 27.

In the body, leptin can circulate as either an unbound adipokine or bound to a soluble-receptor. It is significant to note that lean individuals typically have a majority of bound leptin, while obese individuals tend to have mostly unbound circulating leptin 28. Studies indicate that lower levels of leptin are associated with obesity and overeating, though this has called into question the association between obesity and high leptin levels in humans 26. A decrease in leptin, as occurs in times of starvation or fasting, leads to a reduction in anorexigenic peptides and increases levels of orexigenic peptides, leading to suppression of energy expenses (including sex and thyroid hormones) and increased feeding behavior.

In a healthy individual, leptin primarily functions in regulating appetite and the expenditure of energy (hence its nickname the “satiety hormone”). It is usually expressed in the mammary epithelium, fibroblasts, and skeletal muscles, amongst others, though its expression has also been noted in areas beyond these traditional tissues. Further research modified this definition of leptin's function to inform the brain about energy deficiencies in the body, rather than just regulating appetite. Within WAT, leptin has been shown to inhibit insulin-binding as well as insulin's effects on glucose transport, glucose synthesis, and the production of lipids 27. Some studies have shown that leptin can regulate both enervated and denervated WAT, indicating that it demonstrates both peripheral and central effects on WAT. The type of fat also plays into the secreted levels of leptin, as visceral fat was reported as associated with leptin independent of subcutaneous fat.

There is significant sexual dimorphism when it comes to leptin levels in humans as well; women tend to have significantly higher leptin levels than men 27. Although it was originally thought that this was due to the higher body fat content of women, this notion was quickly disproved, replaced with the theory that leptin production is either stimulated by estrogen or repressed by testosterone and progesterone 27. In addition, there is a significant increase in circulating leptin levels during the stimulation of ovaries, which implicates leptin's role in follicular growth and development 27. This link is further reinforced when an analysis of isolated adipocyte cells indicate that estradiol exposure significantly increased leptin mRNA expression and leptin secretion. This may link leptin to the progression of EC and its role in monitoring the proliferation of tissue in the endometrium as part of the menstrual cycle. However, leptin's relationship with estrogen is still a controversial topic since studies have yielded conflicting results. In terms of menopause, studies show that serum leptin levels are often significantly elevated in obese postmenopausal women and obese premenopausal women in comparison to non-obese premenopausal women 26. In addition, menopause's contribution to the distribution and composition of visceral and subcutaneous adipose tissue within the body may also indirectly affect leptin levels in the aforementioned discussion about adipose tissue's role in estrogen production (in the case that leptin and estrogen are truly linked; again, that point is still under debate).

In terms of obesity in women, leptin has been shown to correlate to an increase in mass accumulation in post-menopausal women, especially those that remained untreated with estradiol or other forms of hormone therapy. These untreated women accumulated much more fat in the trunk area and showed a decline in trunk lean mass in a longitudinal study conducted by researchers at the University of Naples Frederico II 29. Further evidence indicates that leptin levels were significantly higher in women with a BMI exceeding 30 (41.08 leptin levels) compared to those with BMI's less than 25 (5.99 leptin levels) 30. However, it is worth noting that studies tended to find that these changes in leptin levels usually followed the change in weight, rather than predicted them 31.

Leptin may also play a role in the progression of obesity, as it has been shown to be positively correlated with levels of adipose tissue; this is especially concerning since leptin is a pro-inflammatory factor in the body. It induces the production of cytokines in macrophages, which can lead to the development of obesity-related diseases and even cancer in individuals suffering from metabolic syndrome 27. Possible leptin resistance developed during obesity can be attributed to the decrease in transport of leptin across the blood-brain barrier as well as an increase in the inhibitor SOCS3 (typically associated with diet-induced obesity in mice). Studies indicate that leptin production may also be linked to insulin, as it is positively associated with insulin concentration and even increases after administration of insulin. Thus the insulin resistance that often accompanies obesity may come into play 31.

Further research has also found that leptin also plays critical roles in promoting key processes in cancer including metastasis, proliferation, and even drug resistance 28. Cancer cells have been shown to produce leptin and leptin receptor (LEPR), which strongly indicates the existence of a leptin-induced signaling pathway in the proliferation of cancer cells. In several studies, leptin treatment has yielded results indicating leptin as a pro-inflammatory and as a cell cycle progression component. For instance one study concludes that leptin stimulation increased the expression of cyclin D1, a key regulator, in Ishikawa cells 4. In addition, a similar study by another author found that leptin was associated with a decrease in apoptosis in Ishikawa cells; thus it is feasible to consider the notion that leptin may have a role to play in tumor proliferation, growth, and survival. Although leptin expression is higher in other forms of cancer, such as breast cancer and leukemia, EC shows a moderate level of leptin expression compared to other cancers 28, to the point where the expression of leptin and its receptor has been associated with a poorer prognosis (3 years shorter life expectancy) in EC patients. Even within EC, leptin expression shows variability; for instance, expression levels of the receptor Ob-R were higher in poorly and moderately differentiated cells as opposed to well-differentiated EC cells in tissue samples 4. This fluctuation shows we still have much to uncover about the role leptin plays within our bodies and in the progression of tumor growth.

### Visfatin

Visfatin (also known as nicotinamide phosphoribosyltransferase, or Nampt) is an adipokine produced by visceral fat tissue; its expression and plasma levels increase with the onset of obesity 32. It is mainly produced in adipose tissue, though it is also located in skeletal muscle, the liver, bone marrow, and even in lymphocytes. Visfatin plays an additional role of being a PBEF (pre-B-cell enhancing factor), where it is regulated by cytokines that also promote insulin resistance. Its properties as a PBEF are comparable to that of other growth-factor-like cytokines, meaning that visfatin can promote cell proliferation and suppress apoptosis. Within the body, visfatin is characterized by behaving both as an endocrine and paracrine hormone (and occasionally as an autocrine) where it can facilitate the differentiation of local adipose tissue as well as the process of fat deposition. As an endocrine, it may moderate insulin sensitivity in other organs in the body. Since visfatin is primarily produced by visceral adipose deposits, the change in mass of these stores associated with obesity could contribute to metabolic changes, including insulin resistance and abnormal production of adipokines such as visfatin 35. For example, obese individuals typically exhibited higher levels of visfatin, especially those that exhibited IR and MetS parameters 37. In the context of menopause, however, visfatin levels did not seem significantly correlated when controlled for BMI.

One unique characteristic of visfatin is that it does not directly promote insulin-resistance but instead has an effect that mimics that of insulin 33. It can bind to the insulin receptor (IR), a distinct binding site from true insulin, and initiate tyrosine phosphorylation of factors associated normally with insulin binding. By doing so, visfatin can imitate the function of insulin utilizing IR and thus affect glucose uptake, proliferation, and insulin sensitivity. Visfatin's effect is also additive to that of true insulin, and its effect is ultimately mediated by the insulin receptor itself 32. As a mimic of insulin, visfatin promotes the utilization of glucose by adipose tissue and myocytes while suppressing glucose release from liver cells. In addition, visfatin is downregulated by insulin and statins but upregulated by inflammation and hypoxia. Since visfatin has been cited as an inflammatory agent (much like leptin), this piece of information has interesting implications for the self-propagation of visfatin. 36 Others also theorize that visfatin may act through more indirect methods, such as via the brain thermoregulatory system.

Visfatin levels have been associated with many different types of cancer, not just EC 34. Within these different classes of cancers throughout the body, visfatin can act as antiapoptotic, proliferative, pro-angiogenic, and even metastatic factors 35. Universally throughout these cancers, visfatin acted by suppressing apoptotic signaling pathways while enhancing those associated with cellular growth. Although the effects of visfatin vary in severity, these elements were observed in more than twenty types of cancers investigated in the study, located in various parts of the body 35.

With EC, visfatin levels have been identified as a prolific risk factor, alongside other major factors such as age and BMI 31, 33. In a study, it was found that serum visfatin levels were higher in patients with EC compared to control groups. In addition, the study also found that visfatin concentrations were positively correlated with age, fasting insulin, and BMI, among other factors. 31 In the progression of EC, visfatin was found to inhibit the apoptosis of Ishikawa cells and KLE cells by promoting the G1/S phase cell cycle while discouraging apoptosis. Its effects also resulted in a stronger proliferation index in said Ishikawa cells (Ki-67), especially in cases where visfatin was over-abundant 31. In endometrial cells exposed to visfatin, IR, and insulin-receptor substrate as well as other parts of the phosphoinositide 3-kinase and mitogen-activated protein signaling pathways were predominantly expressed. These cells also demonstrated an increase in C-MYC and cyclin D1 as well as decreased caspase-3 expression, an element active in directing apoptosis 31. In addition, visfatin was found to stimulate the DNA synthesis rate of breast cancer cells; it may also augment the expression of genes involved in metastasis. Visfatin's stimulating effects were inhibited alongside the inhibition of P13K and MEK pathways, resulting in lower endometrial cell proliferation and apoptosis 38. Thus, visfatin is a predominant factor in promoting the progression of EC by inducing IR-related signaling pathways in the endometrium. Visfatin was also found to play a major role in enhancing the metastasis progression of EC 34.

### Adiponectin

Adiponectin is a 244-amino acid peptide produced by adipocytes located in adipose tissue (primarily white adipose tissue); other tissue classes such as osteoblasts, skeletal and cardiac myocytes, and endothelial cells can also act as sources of adiponectin. This adipokine directly affects components of the liver, skeletal muscles, and vasculature 38. Adiponectin receptors manifest in two different transmembrane forms: AdipoR1 and AdipoR2 39. Within the bloodstream this protein can exist as three different oligomeric complexes, depending on their molecular weights; each of these forms has distinct properties, such as the lower weights being more closely associated with anti-inflammatory properties while the higher molecular weights facilitate insulin sensitivity. Synthesis and secretion of adiponectin are controlled mainly by substances found in the endoplasmic reticulum, especially when it comes to post-translational modifications. This regulation allows for the secreted molecule to remain stable throughout circulation 38.

In contrast to the previous adipokines discussed, adiponectin acts as an anti-diabetic and anti-inflammatory agent while also increasing sensitivity to insulin (mediated by AMP kinase) 39. This observation led to a surge in adiponectin research in hopes of utilizing its effects to treat metabolic abnormalities and even obesity. For instance, by utilizing fatty acid oxidation in skeletal muscles, improved insulin signal transduction, and increased peripheral tissue sensitivity to insulin, adiponectin plays a vital role in counteracting the effects of insulin-upregulated estrogen 40. Other potential roles that adiponectin is theorized to play is by inhibiting estrogen receptors and vascular endothelium growth, thereby slowing cell proliferation and tumor angiogenesis 41. Adiponectin can also block the proliferative agents of several mitogenic growth factors (including leptin. estradiol) by blocking them from interacting with membrane receptors 43. Adiponectin may also directly inhibit carcinogenesis through triggering AMPK and suppressing phosphatidylinositol 3-kinase (PI3K)/AKT/mTOR signaling; these processes allow adiponectin to up-regulate preventative components such as p53 and Bax while downregulating cyclin D1, c-myc, and other cell cycle progressive factors 45.

Post-menopausal women tended to exhibit significantly lower levels of circulating adiponectin, indicating a correlation with age and/or the sex hormones associated with menopause 43. In fact, in many case studies significantly lower adiponectin levels were correlated with a higher incidence of EC. It is significant to note that lower adiponectin levels were also correlated with higher BMIs, thus reinforcing the role it plays in obesity, despite adiponectin's origin from adipose tissue 42. In the case of obesity, scientists theorize that the way adipose tissue grows and expands in obesity could inhibit adiponectin production and signaling; this is supported by the observation that the receptor expression for adiponectin decreases following the gain of visceral fat 39. This theory is further supported by clinical trials revealing that adiponectin levels increased following weight loss in obese subjects. Lower levels of circulating adiponectin in obese patients correspond to a decrease in insulin sensitivity, leading to increased conversion of glucose and glycogen into fats; these in turn, are taken up by skeletal muscles and thus exacerbate the obese condition 44.

In EC, there exists an inverse relationship between blood adiponectin levels and the risk for developing EC. This may be explained by the previously described anti-tumor and anti-inflammatory properties adiponectin has shown to exhibit, from inhibiting factors that would promote cell growth and tumor progress to outright downregulating intracellular factors that accelerate cell cycles and upregulating those that check cell cycles, as described above. Lowered adiponectin levels, especially in post-menopause and in obesity, represent weaker defenses against the development of EC; obesity's adverse effects on adiponectin concentration would only amplify this condition. However, this field is still relatively controversial due to how little is known about the effect adiponectin has on other organs. For instance, in some forms of cancer such as breast cancer, adiponectin is shown to have potential anti-apoptosis effects that actually increase the risk of breast cancer. As such a deeper understanding of this field is paramount before concrete steps can be taken to implement this potent contender into treatments 46.

## Conclusion

Major hormones that shift in concentration in obesity (especially menopause-induced obesity) include insulin, estrogen, and adipokines. These have direct impacts on the risk and progression of endometrial cancer. The purpose of this review was to establish the exact relationships amongst these factors and endometrial cancer while tracing the causes of their changes in concentration back to obesity and menopause. All of these hormones have very definite relationships with both obesity and endometrial cancer and are worth investigating further in the future; this paper served as a brief overview of their roles in the context of a post-menopausal woman's body. Research in this field is of rising concern as the incidence of both obesity and endometrial cancer increase in occurrence currently; thus, it is important to begin looking for preventative measures, possible by investigating ways to counteract or even control these hormone levels.
